# Combination of celecoxib with percutaneous radiotherapy in patients with localised prostate cancer – a phase I study

**DOI:** 10.1186/1748-717X-1-9

**Published:** 2006-04-10

**Authors:** U Ganswindt, W Budach, V Jendrossek, G Becker, M Bamberg, C Belka

**Affiliations:** 1 CCC Tübingen, Centre for Genitourinary Oncology, Department of Radiation Oncology, University of Tübingen, Tübingen, Germany; 2 Department of Radiation Oncology, University of Düsseldorf, Düsseldorf, Germany; 3 Department of Radiation Oncology, Klinik am Eichert, Göppingen, Germany

## Abstract

**Background:**

Current approaches for the improvement of bNED for prostate cancer patients treated with radiotherapy mainly focus on dose escalation. However molecularly targeted approaches may also turn out to be of value. In this regard cyclooxygenase (COX)-2 inhibitors have been shown to exert some anti-tumour activities in human prostate cancer *in vivo *and *in vitro*. Although *in vitro *data indicated that the combination of COX-2 inhibition and radiation was not associated with an increased toxicity, we performed a phase I trial using high dose celecoxib together with percutaneous radiation therapy.

**Methods:**

In order to rule out any increases of more than 20% incidence for a given side effect level 22 patients were included in the trial. Celecoxib was given 400 mg twice daily with onset of the radiation treatment. Risk adapted radiation doses were between 70 and 74 Gy standard fractionation. RTOG based gastrointestinal (GI) and genitourinary (GU) acute toxicity scoring was performed weekly during radiation therapy, at six weeks after therapy and three month after completing radiation treatment.

**Results:**

Generally no major increase in the level and incidence of side effects potentially caused by the combined treatment was observed. In two cases a generalised skin rash occurred which immediately resolved upon discontinuation of the drug. No grade 3 and 4 toxicity was seen. Maximal GI toxicity grade 1 and 2 was observed in 85% and 10%, respectively. In terms of GU toxicity 80 % of the patients experienced a grade 1 toxicity and 10 % had grade 2 symptoms.

**Conclusion:**

The combination of irradiation to the prostate with concurrent high dose celecoxib was not associated with an increased level of side effects.

## Background

Prostate cancer is the most common malignant tumour in men. At present, approximately 200.000 new diseases are diagnosed per year in the USA leading to the death of more than 30.000 patients. Due to the increased use of PSA screening the number of patients diagnosed in localised disease is rising strongly. Radical prostatectomy, percutaneous radiotherapy and interstitial radiation methods are available for curative treatment of localised stages. Due to a lack of randomised studies, the optimal treatment is still unclear. Based on the available data, however it seems likely that all given methods are more or less equivalent in terms of tumour control. Side effects in the rectum predominate with percutaneous radiotherapy, while mainly impotence and incontinence are seen after prostatectomy [1].

Nevertheless, a crucial problem is still unsolved. The long natural history of prostate cancer makes it difficult to determine which type of local therapy is best in men with life expectancies longer than 8–10 years at diagnosis. In this regard, long-term follow-up data with overall survival as endpoint and meticulous determination of side effects will finally answer the question whether there is an optimal therapy for localised prostate cancer.

Local control rates (defined as biochemically relapse-free five-year survival) between ~ 50 and ~ 90% can be achieved with percutaneous irradiation for localised stages. All available data indicate the existence of a clear dose-effect relationships for pathological control as well as bNED [2-9]. Hence, strategies for increasing the radiation dose are an important goal when trying to optimise the outcomes after radiotherapy. In order to increase the dose, intensity-modulated radiotherapy or particle based therapy approaches are currently under investigation [10-16].

In addition to an increased radiation dose, the blockade of testosterone action was found to be an effective measure for improved radiation treatment results [17-20].

To further optimise the efficacy of radiation treatments, molecular targeted approaches are currently under investigation [21].

Of special importance are drugs targeting tyrosine receptor associated kinase pathways (EGF-R, VEGF-R, IGF-R) downstream kinase molecules, and cell death signalling pathways [22-26]. Beside this, numerous reports underline the importance of prostaglandin signalling during cancer development and growth [27-30]. In addition it has been suggested that the modulation of prostaglandin generation may alter treatment responses towards chemotherapy and radiation [31-34].

A key enzyme involved in prostaglandin synthesis is the inducible cyclooxygenase-2 molecule which is frequently found to be overexpressed in human cancer cells, whereas in non-malignant tissues COX-2 is predominantly found in association with inflammatory processes [35-37]. The development of selective COX-2-inhibitors thus theoretically allows a tumour specific response modulation.

Based on these findings, COX-2 inhibitors were shown to be effective in patients with FAP, where the number of polyps is strongly reduced when patients received 2 × 400 mg celecoxib per day. Importantly lower doses had less effects on the development of adenomas [38].

Although the inhibition of the COX-2 enzyme by celecoxib is important for the understanding of its efficacy, several data suggest that celecoxib may exert non-COX-related effects in cancer cells [39-43]. In this regard, Waskewich [44] showed that celecoxib induces clonogenic cell kill with similar IC50 values irrespectively of the COX-2 expression status. Although the mechanisms of the non-COX-dependent action of celecoxib are not completely understood, several data suggest that they are related to the fact that celecoxib triggers a new apoptosis mitochondrial apoptosis pathway or interferes with PKB AKT signalling. Especially the pro-apoptotic effect was found to require doses higher than needed for an inhibition of the regular target enzyme. In this regard the data on FAP suppression are important, since there was a clear dose response relationship above the anti-inflammatory dose level.

Although celecoxib seems to be active alone, several groups provided evidence that the drug is considerably more effective when combined with a second anti-tumour treatment option. A comparative study in animals showed that the combination of radiotherapy with COX-2 inhibitors produces a clearly improved response rate when compared to radiotherapy alone. The TCD 50 values (FSA sarcoma xenograft) were found to be halved in case of a combined treatment [45,46].

Antitumour activities of COX-2 inhibitors have been shown for various human malignant tissues including colorectal [38,47,48], breast [29,49], non small cell lung [50,51] and other epithelial cancers [42,52-54].

Therefore the role of the combination of an COX-2 inhibitor with other treatment modalities has mainly been tested in lung cancer, cervical cancer, head and neck cancer and colorectal cancers.

Several lines of evidence point to a role of COX-2 inhibition as treatment approach for prostate cancer [39,43,55-61] (table 1). Histological analysis of prostate carcinoma cells revealed an overexpression of COX-2 in tumour tissue when compared to normal prostate stroma or benign prostatic hyperplasia [59].

**Table 1:** Overview on the available mechanistic data regarding the activity of coxibes in prostate cancer

Cancer Type	Treatment	Investigation	Results	Reference
LNCaP PC 3	Celecoxib	In vitro	Increased cell death/apoptosis	Kamijo 2001
PC 3	Celecoxib	In vitro/Xenograft	G1 block/reduced DNA synthesis/growth inhibition by COX-2 independent mechanism	Patel 1999
LNCaP PC 3	Celecoxib	In vitro	Growth inhibition	Srinath 2003
LNCaP PC 3	Celecoxib	In vitro	Induction of apoptosis by blocking Akt activation independently of Bcl-2	Hsu 2000
PC 3	Celecoxib+ radiation	In vitro	Up-regulation of COX-2, elevated PGE_2 _levels after irradiation	Steinauer 2000
LNCaP PC3 DU-145	Celecoxib+ radiation	In vitro	Bax-independent pro-apoptotic effect of Celecoxib	Handrick 2004
LNCaP DU-145 PC-3ML	Celecoxib+ COL-3/Docetaxel	In vitro/Xenograft	Augmentation of chemotherapeutic drug-induced apoptosis by activation of caspase 3 and 9	Dandekar 2005

COX-2 contributes to the proliferation of prostate cancer cells, while COX-2 inhibitors were clearly shown to inhibit proliferation and to induce apoptosis [60].

In the setting of hormone refractory prostate cancer the application of celecoxib in patients was associated with some partial PSA responses [62]. Likewise in patients with biochemical relapse after definitive therapy a significant inhibition of serum PSA levels 3 months after treatment with celecoxib was observed [63].

Furthermore, it could be shown *in vitro *that irradiation of PC-3 cells triggers an increase in COX-2 expression [64]. In own studies, the combination of celecoxib with ionising radiation revealed an additive effect on cell kill in PC-3 and DU-145 cells [65].

Based on murine data the combination of celecoxib with irradiation seems not critical regarding toxicity [45]. However, recent clinical data suggest that at least in an multi-modality setting the addition of celecoxib to a chemoradiotherapy protocol may be associated with increased toxicity rates [66].

In order to rule out any safety concerns of a combination of celecoxib with irradiation we prospectively determined the toxicity of such an combination in prostate cancer using the highest Food and Drug Administration-approved dose of 800 mg celecoxib daily.

## Methods

### Aim of the study

Aim of the study was to determine the acute toxicity of a celecoxib administration during percutaneous radiotherapy of localised prostate cancer. The primary endpoint of the study was the incidence of acute toxicity (up to three months after therapy).

### Inclusion and exclusion criteria

Patients with histologically proven prostate cancer, stages cT1-cT3 cN0 cM0, G1-3, PSA ≤ 20 ng/ml, age up to 75 years and Karnofsky Index ≥ 80 %, were included after providing informed consent. Further inclusion criteria were normal levels of hemoglobin, leukocytes, platelets, creatinine, urea, GGT, AP, AST, ALT, bilirubine, creatinine clearance > 50 ml/min and no other clinically leading secondary disease. Any other NSAIDs were not allowed with the exception of acetylsalicylic acid at a cardioprotective dose. Patients after transurethral resection or prostatectomy and patients with a known contraindication (e.g. gastric ulcer) or allergy to COX-2 inhibitors were excluded. Further exclusion criteria were severe heart, cardiovascular, liver, renal, inflammatory intestinal or blood coagulation disorders, collagenoses, former irradiation of the prostate, secondary malignancies (exception non-melanotic skin cancer) and regular intake of lithium or fluconazole.

### Staging examinations

The pre-therapeutic staging examinations included the initial PSA value, biopsy with histological confirmation and statement of the grading or Gleason score, rectal digital examination, transrectal endosonography and at least pelvic sonography, alternatively computed tomography (CT) or magnetic resonance imaging (MRI), to evaluate the lymph nodes. At PSA levels > 10 ng/ml a bone scintigraphy was mandatory.

### Treatment course

All patients were treated with celecoxib 400 mg twice daily in an open-label, unblinded trial during the entire series of radiation. The intake of celecoxib was started on the first day of radiotherapy, continued also on radiation-free days (e.g. weekends) and stopped on the last day of radiotherapy. Celecoxib medication was discontinued, if a patient developed ≥ grade 3 toxicity. The percutaneous radiotherapy was planned with a three-dimensional (3D) radiation planning system based on computed tomographies in supine position. A rectal balloon filled with 40 ml of air was used in order to spare the posterior wall of the rectum and for fixation of the prostate [67]. An additional 3D radiation planning without the rectal balloon was performed simultaneously for use in case of non-tolerance of the balloon. We used a conformal, isocentric 4-field technique with 15 MV photons. Target volume and dose concept depended on a risk classification based on the prognostic factors stage, grading and initial PSA level. The patients received 5 × 2.0 Gy per week up to 70.0 Gy and 74.0 Gy cumulative dose, respectively. The planning target volume (PTV) included the risk dependent clinical target volumes (table 2) with a safety margin of 10 mm (with rectal balloon) and 12 mm (without balloon), respectively. The patients with a high risk of relapse treated with 74.0 Gy cumulative dose received a boost of 8 Gy with a dorsal safety margin of 5 mm followed by 66 Gy as described above. As organ at risk the whole rectum from anal sphincter to the location where the rectum turned horizontally into the sigmoid colon was defined. The given radiotherapy doses were prescribed in line with ICRU Report No 50 and the given volumes complied with the definitions of ICRU Report No 62. Additional hormone therapy could be freely used as part of the study.

**Table 2:** Target volume and dose concept depending upon stage, grading and PSA

Low risk: white	Medium risk: light grey	High risk: dark grey	
**Stage**	≤ cT2a	≤ cT2c	cT3
**PSA**	≤ 10	< 20	< 20
**G 1 Gleason 2–3**	Prostate	Prostate & base of seminal vesicles 70 Gy	Prostate & base of seminal vesicles & visible tumour 74 Gy
**G 2 Gleason 4–6**	Prostate	Prostate & base of seminal vesicles 70 Gy	Prostate & base of seminal vesicles & visible tumour 74 Gy
**G 3 Gleason > 6**	Prostate & base of seminal vesicles 70 Gy	Prostate & base of seminal vesicles 70 Gy	Prostate & base of seminal vesicles & visible tumour 74 Gy

### Laboratory measurements

The creatinine clearance was examined prior to inclusion into the study. Prior to treatment start, at week 2, 4, 6 of the combined therapy and 3 months after the end of treatment blood samples were taken. The measurements included a blood count, coagulation parameters and serum levels of electrolytes, creatinine, urea, GGT, AP, AST, ALT and total bilirubine. PSA levels were measured prior to treatment start and after three months.

### Measurement of acute toxicity

Acute toxicity according to RTOG criteria (gastrointestinal, genitourinary) was acquired at least once weekly during the 7–8 week series of radiation treatment, 6 weeks and 3 months after treatment. Beside the clinical examination documented on case report forms we used a standardised questionnaire that had to be filled by the patients at the same time. Beside acute gastrointestinal and genitourinary toxicity according to RTOG criteria any other acute toxicity was described on the case report form. Late toxicity is further ascertained as part of the radiotherapeutic follow-up examination outside the study once a year.

### Criteria for discontinuation/statistics

The acute toxicity data published by Storey et al. [68] with cumulative doses from 70 to 78 Gy were the reference basis for the toxicity to be anticipated in our study. The study was powered to exclude an > 20% increase in the incidence of grade 3 and 4 acute GI and GU toxicity. Derived from these conditions the following criteria to close the study prematurely were defined: If no grade 4 acute toxicity would occur in 20 patients, the 95% confidence interval is 0 to 16.8%. The study would then be discontinued, because at 95% safety acute toxicity of 20% or more could be ruled out. If exactly one grade 4 acute toxicity would occur, the 95% confidence interval is 0.1 to 24.9%. The sample size has then to be increased by further 15 patients. If there would remain just one case of grade 4 acute toxicity, the 95% confidence interval is 0.1 to 14.9%, with one further case 0.7 to 19,2%, i.e. it would not include the critical value of 20%. If two cases of grade 4 toxicity would occur in the first 20 patients, further 15 patients would be recruited. In case of no further grade 3 or 4 toxicity, the 95% confidence interval is 0.7 to 19.2%. If at least three cases of grade 4 toxicity would occur in just the first 20 patients, the study would be discontinued. Even when treating additional 15 patients the predefined acceptable toxicity level would have been exceeded.

## Results

### Patient characteristics

From 06/2003 to 07/2004 22 patients were included into the study. All 22 patients completed the radiotherapy without treatment break. In all cases the intake of celecoxib started at the first day of radiotherapy in the morning. Within 2 weeks after commencing treatment 2 of the 22 included patients displayed a general exanthema with pruritus. Medication was stopped immediately and the skin rash resolved completely afterwards. Therefore we assumed that this reaction was a drug allergy. Both patients were excluded from the trial. The other 20 patients completed the treatment according to the study protocol with 400 mg celecoxib twice daily. 5 patients received 74 Gy cumulative dose, 14 patients received 70 Gy cumulative dose and 1 patient was treated with 72 Gy. Median age was 67 years (range 49 – 74 years); median initial PSA-level was 8 ng/ml (range 2,4 – 18,3 ng/ml). 14 patients received hormone ablative therapy (table 3), mostly started before and continued concurrently to radiotherapy. The rectal balloon was tolerated well, 2 patients' radiotherapy treatment was continued without rectal balloon after 40 and 46 Gy, respectively. The resulting dose-volume-histograms of the rectum are shown for all patients in figure 4.

**Table 3:** Patients Characteristics

Characteristics	No. of patients
Age	
< 67	10
> 67	10
T-Stage	
1c – 2a	12
2b – 2c	6
3	2
Initial PSA	
≤ 10 ng/ml	13
> 10 ng/ml	7
Gleason Score	
≤ 6	13
≥ 7	7
Hormonal ablation	
Yes	14
No	6

### Acute gastrointestinal and genitourinary toxicity

**Figure 1:**
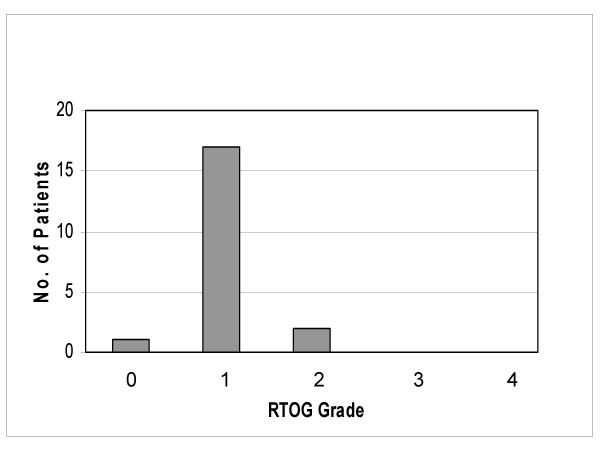
Acute gastrointestinal toxicity (RTOG).

No gastrointestinal or genitourinary acute toxicity grade 3 or 4 (RTOG) occurred. Thus we finished patient recruitment after complete treatment of 20 patients. 17 of 20 patients showed a gastrointestinal acute toxicity grade 1. 2 of 20 patients showed a gastrointestinal acute toxicity grade 2. Most frequent grade 1 symptom was mild rectal discomfort. Among he 2 patients with grade 2 gastrointestinal toxicity 1 patient had diarrhoea and the other patient required mild analgetics for his rectal symptoms (figure 1).

**Figure 2:**
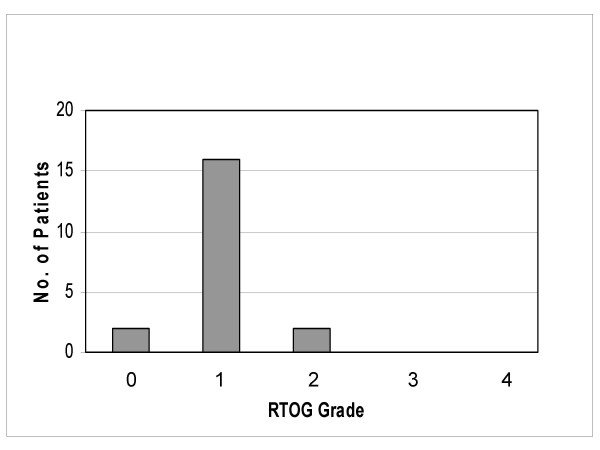
Acute genitourinary toxicity (RTOG).

In 16 of 20 patients we observed a genitourinary acute toxicity grade 1, in 2 of 20 patients a genitourinary acute toxicity grade 2. Most frequent grade 1 symptom was slight dysuria. Among the 2 patients with grade 2 genitourinary toxicity 1 patient had bladder spasms, the other patient presented with a bacterial cystitis 3 weeks after radiotherapy, which completely resolved after treatment with adequate antibiotics (figure 2).

### Other toxicity

**Figure 3:**
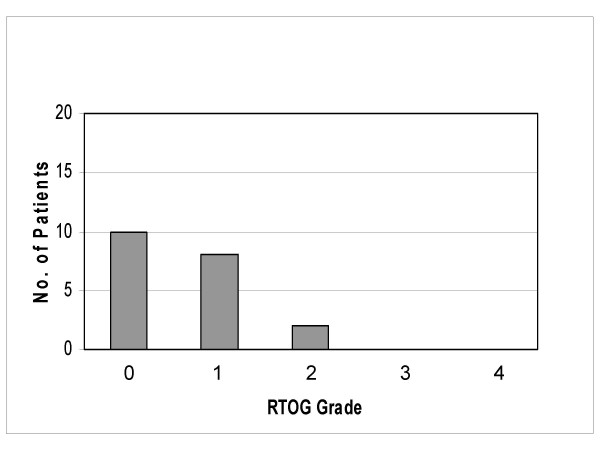
Acute skin toxicity (RTOG).

Considering the acute skin toxicity we observed 2 patients with a grade 2 toxicity (circumscribed moist desquamation measuring 1–2 cm per patient), 8 patients with a grade 1 toxicity and 10 patients with no toxicity at all (figure 3). Based on the clinical examinations, the taken blood samples and the questionnaires filled by the patients we observed no other acute toxicity. With exception of the 2 patients described above who developed a drug allergic reaction no cardiovascular, gastric, renal, hepatic or bone marrow side effect of celecoxib occurred.

**Figure 4:**
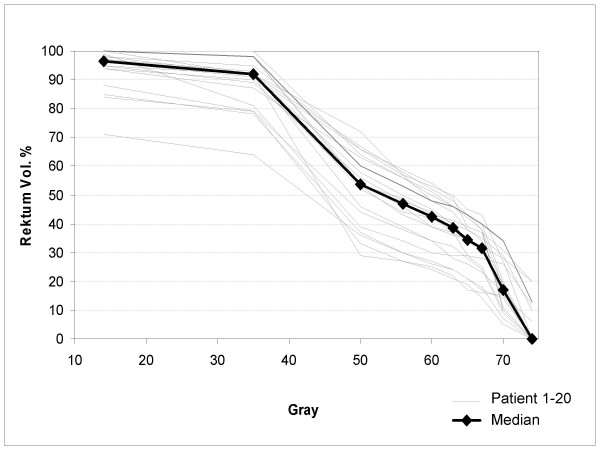
Dose-volume-histograms of the rectum.

## Discussion

Several approaches for the improvement of bNED in the radiotherapeutic treatment of localised prostate cancer were tested. Current strategies mainly focus on dose escalation. In this regard, new radiation technologies for example IMRT allow the application of high radiation doses without increasing the toxicity. In addition, the combination with hormonal treatment has been proven to be suitable to increase local control and biochemical relapse-free interval rates. The results of four major trials [18-20], [69-72] revealed that a combined treatment is advantageous for intermediate and high risk patients. Patients with an intermediate risk profile benefit both from radiation dose escalation and additional hormonal treatment, even if there is no clear cut recommendation regarding starting time and duration of hormonal treatment for intermediate risk patients. However molecularly targeted approaches may also turn out to be of value. In this regard, preclinical studies suggest that COX-2 inhibitors have an certain anti-tumour activity when given alone and are even more active when combined with classical anti-tumour treatment.

In case of prostate cancer, a clear dose response relationship exists for the endpoint local control and bNED especially in patients with a low or intermediate risk profile. Although *in vitro *data indicated that there is no increased toxicity when COX-2 inhibitors are combined [45] with radiation, there are few clinical data concerning the toxicity of a combined treatment. The aim of our prospective trial was to determine the acute toxicity of a simultaneous celecoxib and radiotherapy application.

An > 20% increase in the incidence of grade 3 and 4 acute GI and GU toxicity could be excluded. We did not observe any grade 3 or 4 toxicity. With exception of 2 patients with a drug allergic reaction no systemic side effects were obvious. The cumulative rates of grade 0 – 2 toxicities are in the same range as already documented by others [14,16,68,73,74]. However, we observed a larger proportion of grade 1 toxicities (gastrointestinal and genitourinary). This finding may simply reflect a certain lack of precision for the definition of grade 1 effects using the RTOG criteria, allowing inaccuracies when comparing patient sets from different investigators.

Although tested in a rather small cohort, our prospective data suggest that it is save to combine the highest FDA approved dose of celecoxib with intermediate radiation dose concepts for prostate cancer. This observation is in keeping with our clinical impression that, despite a widespread clinical use of coxibes as pain relievers in the past, no major problems occurred.

However, our data do not allow an incautious use of coxibes in other clinical settings. This holds especially true when more complex regimes are taken into account. In this regard, the analysis of the early toxicity of RTOG 0128 treatment arm testing a combination of pelvic radiotherapy, 5-FU, cisplatin and celecoxib for advance cervical cancer revealed major GI toxicity in ~ 50% of the treated patients [66]. Similarly, a clinical phase I trial at the M.D. Anderson Cancer Center in patients with pancreatic cancer has revealed more toxicity when celecoxib was added to a chemoradiation with gemcitabine [75]. Thus a meticulous toxicity testing should be performed when ever attempting to combine celecoxib with radiation alone and more importantly, when additional cytotoxic drugs are applied.

A different picture emerges from some other phase I/II trials showing that celecoxib combined with radiation or chemoradiation is safe and well tolerated. Liao et al. [76] tested escalated (200–800 mg daily) celecoxib doses combined with thoracic radiotherapy in patients with inoperable NSCLC and showed safe administration of 800 mg celecoxib daily and encouraging preliminary outcome results. An additional phase I/II trial concerning 27 patients with brain metastases treated with radiation and celecoxib [77] confirmed the feasibility and safety. Govindan et al. [78] treated patients with oesophageal cancer with cisplatin, 5-FU and celecoxib and concluded, that the addition of celecoxib to chemoradiation is well tolerated. The results of ongoing phase I and phase I/II trials combining celecoxib with either radiation or radiation plus chemotherapy have to be expected.

Although initially announced to be pain medications with an low and optimal toxicity profile, severe concerns regarding the safety of the coxibes as drug family came up when an increased rate of non-fatal cardiac events was observed in patients treated with rofecoxib for rheumatic disorders over longer periods of time [79]. Unfortunately, these observations seem to have discredited the use of coxibes over a short term as putative anti-neoplastic agents. Up to now no data are available on a potential increase in cardiac and vessel related side effect when coxibes are used over a short time period and in higher doses. Since there are no data available to finally judge the value of coxibes in oncology we find it not justified to suspend clinical testing of coxibes in an oncology setting based on the results from long term use in rheumatology. This is even more underlined by the fact that the comparatively high toxicities are acceptable for anti-neoplastic drugs when compared with simple pain relievers.

## Conclusion

In comparison with published data the toxicity of a combination of high dose celecoxib and radiotherapy for prostate cancer is not increased. Further phase II and III testing is required for efficacy testing.

## Abbreviations

AP Alkaline phosphatase

AST Aspartat-Aminotransferase

ALT Alanin-Aminotransferase

BNED Biochemical no evidence of disease

CT Computed tomography

CTV Clinical target volume

EGF-R Epidermal growth factor receptor

FAP Familial adenomatous polyposis

5-FU 5-Fluorouracil

G Grading

GGT Gamma-Glutamyltransferase

Gy Gray

IC 50 value Inhibitory concentration of 50 %

ICRU International Commission on Radiation Units and Measurement

IGF-R Insulin-like growth factor receptor

mg Milligram

MRI Magnetic resonance imaging

NSAID Non steroidal anti-inflammatory drugs

NSCLC Non-small-cell- lung-cancer

PSA Prostate specific antigen

PTV Planning target volume

RTOG Radiation Therapy Oncology Group

TNM Tumour/nodal/metastases stage

TCD 50 Radiation dose yielding 50 % tumour cure

VEGF-R Vascular endothelial growth factor receptor

## Competing interests

The author(s) declare that they have no competing interests.

## Authors' contributions

WB, CB & UG, VJ planned, coordinated and conducted the study. UG analysed the data. UG & CB prepared the manuscript. Medical care was covered by UG, CB, WB & MB. All authors read and approved the final manuscript.
